# Repurposing PFM01 as a novel antibacterial agent against *Staphylococcus aureus*

**DOI:** 10.1128/spectrum.01372-26

**Published:** 2026-10-01

**Authors:** Congcong Li, Xuancheng Huang, Kewei Fan, Jintuan Lin, Yong Xiang, Bing Bai, Haigang Zhang, Zhijian Yu, Tieying Hou

**Affiliations:** 1 School of Basic Medical Sciences, Shenzhen University Medical School, Shenzhen University 617007, Shenzhen, China; 2 Department of Infectious Diseases and Shenzhen Key Laboratory for Endogenous Infections, Shenzhen Nanshan People's Hospital, Affiliated Nanshan Hospital of Shenzhen University, Shenzhen, China; 3 School of Pharmacy, Shenzhen University Medical School, Shenzhen University 620600, Shenzhen, China; 4 Department of Critical Care Medicine, Shenzhen Nanshan People's Hospital, Shenzhen University Medical School 481870, Shenzhen, China; 5 Marketing Department of Guangzhou Branch of Varian Medical Equipment Trading (Beijing) Corp, Guangzhou, China; Universita degli Studi di Bari Aldo Moro, Bari, Italy

**Keywords:** *Staphylococcus aureus*, PFM01, drug repurposing, antibacterial activity, biofilm, membrane disruption

## Abstract

*Staphylococcus aureus* infections pose a significant threat to global human health, particularly with the widespread transmission of methicillin-resistant *S. aureus* (MRSA) strains, which has created an urgent need for the development of new antimicrobial agents. PFM01 is an N-alkylated derivative of Mirin, an MRE11 endonuclease inhibitor. It was initially discovered to block the repair of double-strand DNA breaks and demonstrated preliminary anti-tumor potential; however, its antimicrobial activity had not yet been explored. Here, the anti-*S*. *aureus* activity of PFM01 was determined by broth microdilution minimum inhibitory concentration (MIC) testing and time-kill kinetics. The *in vitro* cytotoxicity was assessed by CCK-8 assay and hemolysis test. The potential mechanisms were systematically investigated by whole-genome sequencing, drug affinity responsive target stability (DARTS), and molecular docking analyses. PFM01 exhibited potent antibacterial activity against Gram-positive bacteria (including *S. aureus*, *Enterococcus faecalis*, *Enterococcus faecium*, and *Streptococcus lactis*), with MIC_50_ values ranging from 6.25 to 12.5 μM (1.83–3.67 μg/mL). Time-kill curve analysis confirmed its bactericidal capacity against free-floating *S. aureus* (including bacteria in the logarithmic growth phase and stationary phase-retained cells). Furthermore, PFM01 inhibited *S. aureus* biofilm formation in a concentration-dependent manner and possesses the ability to remove mature biofilms. *In vitro* cellular safety tests of PFM01 indicated low cytotoxicity and no significant hemolytic activity. Moreover, PFM01 altered the membrane permeability and potential of *S. aureus*, and its antibacterial activity was counteracted by the exogenous phospholipids. Whole-genome sequencing identified genetic mutations in the *rapZ*, *mupR*, *mprF,* and *clpX* genes of PFM01-resistant *S. aureus* strains; however, the antibacterial activity of PFM01 against *mprF* and *clpX* knockout *S. aureus* strains showed no significant change compared with wild-type strains. Proteomic analysis further revealed that PFM01 primarily might interfere with purine metabolism, amino acid biosynthesis, and the phosphotransferase system. DARTS experiments suggested that glycerol-3-phosphate cytidylyltransferase (TarD) might be a potential target, and molecular docking results further confirmed a stable interaction between PFM01 and TarD (binding energy: −7.4 kcal/mol). In summary, PFM01 may exert its antibacterial effect by disrupting the bacterial cell membrane structure and targeting TarD, and holds promise as a therapeutic agent for *S. aureus* infections.

**IMPORTANCE:**

This study confirms that PFM01 has a broad and strong antibacterial effect against Gram-positive bacteria while also being biologically safe. The antimicrobial mechanism of PFM01 is closely associated with TarD. The advantages of low cytotoxicity against host cells and reduced hemolysis toward red cells were further demonstrated. As a promising repurposed antimicrobial candidate, PFM01 shows potential application for the treatment of infections caused by multidrug-resistant *S. aureus*.

## INTRODUCTION

*Staphylococcus aureus* is a Gram-positive bacterium commonly found on the skin and mucous membranes of humans. Under certain conditions, it can become a highly pathogenic pathogen, causing a wide range of infections, from minor skin infections, cellulitis, endocarditis, and pneumonia to life-threatening sepsis (1).

The complex pathogenic mechanisms of *S. aureus* infections make it difficult to control the spread of this bacterium. One typical mechanism of *S. aureus* pathogenicity involves the rapid production of various virulence factors and toxins, which can directly damage host tissues or exert their invasive effects through immune-mediated mechanisms (2, 3). Furthermore, *S. aureus* is capable of forming the biofilms, which shield itself from the inhibitory effects of the antimicrobial agents and immune clearance. Until now, the commonly used antibiotics have limited efficacy in eradicating mature biofilms of Gram-positive bacteria (4).

Antibiotic resistance in *S. aureus* increases the difficulty of clinical treatment. Mounting reports have confirmed the rapid development and dissemination of the acquired antibiotic resistance in *S. aureus* clinical isolates in recent years (2). For example, methicillin-susceptible *S. aureus* (MSSA) has gradually developed the intractable resistance or non-susceptibility toward the commonly used antimicrobial agents, such as penicillin, cephalosporins, and macrolides, etc. (5, 6). It is worth noting that, globally, an increasing number of MRSA strains are reported with acquired resistance to the last-resort antibiotics such as vancomycin (Van), daptomycin, linezolid, and tigecycline (7, 8). The widespread transmission of MRSA poses a major public health crisis, and the increase of the intractable *S. aureus* infections with multidrug resistance gradually led to prolonged illness, increased healthcare costs, and higher mortality rates in the clinics (9, 10). Due to the limited selection of effective antibiotics, the antimicrobial treatment of multidrug-resistant *S. aureus* infections poses significant challenges in clinical practice (11). Consequently, there is an urgent need to develop novel anti-*S*. *aureus* agents capable of killing bacterial cells and eradicating biofilms to improve clinical outcomes in the antimicrobial treatment of such infections. The bacterial cell membranes act as a major physical barrier against antibiotic penetration and have been widely considered as the rational target for the development of novel antimicrobial agents (12, 13). Interestingly, the phospholipids in the outer membrane of bacterial cells sometimes can interfere with membrane-targeting antibiotics, which compete for the crosslinking binding sites of these phospholipid molecules and disrupt the membrane integrity (14). Consequently, lipid homeostasis can be disrupted by targeting the bacterial membrane, and bacterial membrane phospholipids have emerged as attractive therapeutic targets that might overcome the present resistance mechanisms.

Against this backdrop, drug repurposing has emerged as a promising and highly effective strategy (15). It refers to the identification of new therapeutic uses for already established compounds. PFM01 is an N-alkylated derivative of Mirin, initially identified as an inhibitor of the MRE11 nuclease (16, 17). This enzyme is a key component in the DNA double-strand break repair (DSBR) pathway, which includes non-homologous end-joining (NHEJ) and homologous recombination (HR) processes (18). PFM01 has been studied as a potential anticancer agent by interfering with DNA repair mechanisms in tumor cells (19). However, its potential antimicrobial properties have not yet been fully explored.

## RESULTS

### Antibacterial activity of PFM01 against *S. aureus*

**Fig 1:**
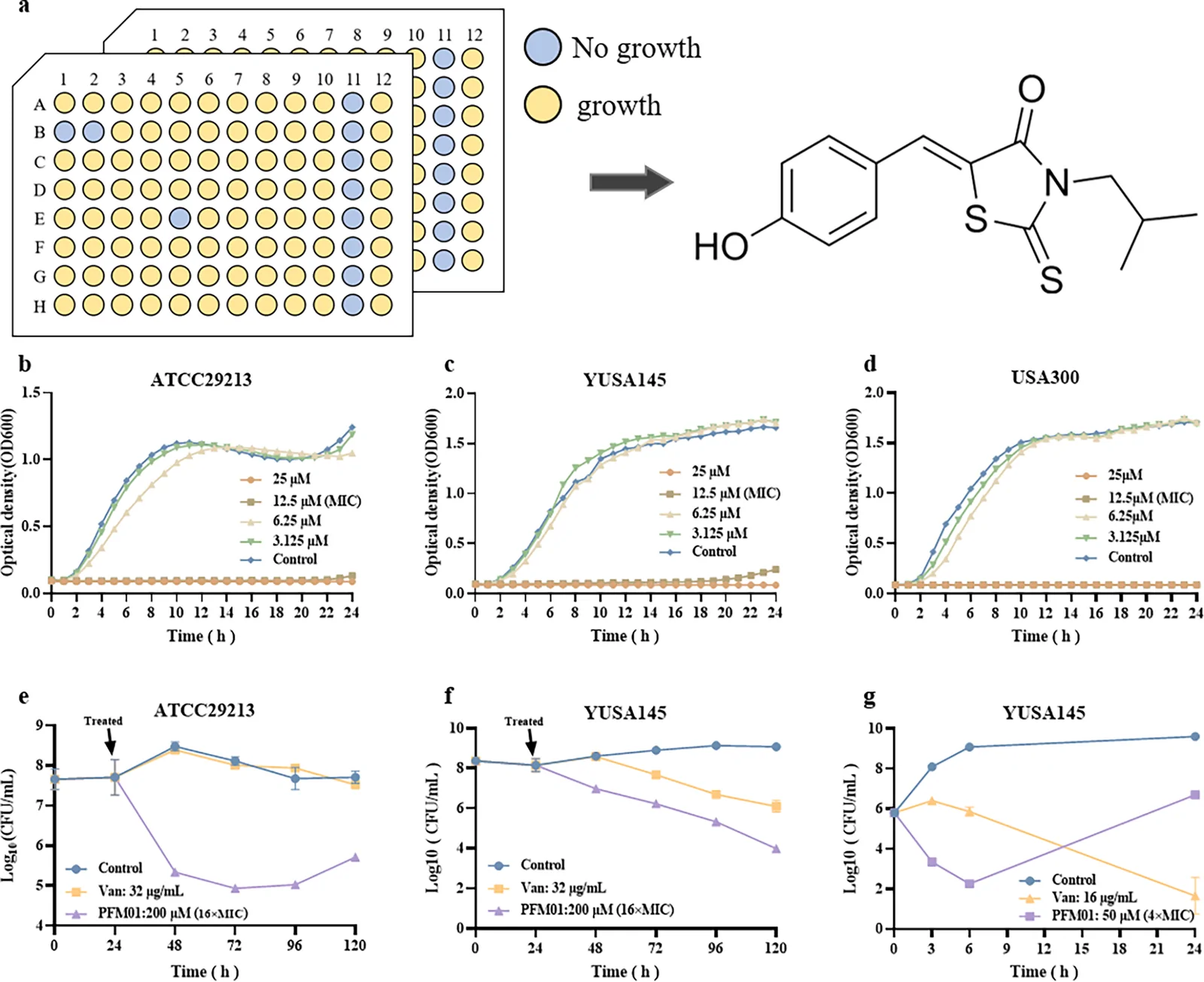
The inhibition of *S. aureus* growth by PFM01 through automatic growth curve and time-kill curve. (**a**) High-throughput screening of antibacterial compounds and the chemical structure of PFM01. *S. aureus* strains SA113 and USA300 were used as target organisms. Test compounds were added to columns 1 to 10 (odd columns: 50 μM; even columns: 12.5 μM), with the same compound in adjacent rows; column 11 was a blank control, and column 12 was a positive control. (**b–d**) Inhibition of planktonic cell growth after 24-h treatment with PFM01 at indicated concentrations against clinical isolates MSSA ATCC 29213 (**b**), MRSA YUSA145 (**c**), and USA300 (**d**). Bacterial density is determined by measuring OD_600_. (**e and f**) Time-kill assay of PFM01 against stationary-phase cells of MSSA ATCC 29213 (**e**) and MRSA YUSA145 (**f**). Van (32 μg/mL) serves as the positive control. (**g**) Time-kill curve of PFM01 against log-phase cells of MRSA YUSA145. Van (16 μg/mL) serves as the positive control. For all time-kill assays, groups without PFM01 treatment are used as negative controls. *n* = 3, and data are presented as mean ± SD.

In this study, high-throughput screening on a library of 1,000 compounds at two fixed concentrations (12.5 and 50 μM) was performed using *S. aureus* strains SA113 and USA300 as the target organisms. Compounds showing activity at 12.5 μM were selected as initial hits, and PFM01, an N-alkylated Mirin derivative, was identified as a promising candidate for further study based on its antibacterial potency (Fig. 1a). Subsequently, to evaluate its potential for *in vivo* application, we systematically assessed the inhibitory activity of PFM01 against a range of clinically isolated Gram-positive bacteria, comprising a total of 80 strains of *S. aureus* (including 41 MSSA and 39 MRSA strains), 22 strains of *Staphylococcus epidermidis*, 19 strains of *Enterococcus faecalis*, and 21 strains of *Enterococcus faecium*. Based on the MIC distribution (Table 1), PFM01 exhibited MIC_50_ values of 12.5 μM (equivalent to 3.67 μg/mL) for MSSA, MRSA, *E. faecium*, and *E. faecalis*, and 6.25 μM (equivalent to 1.83 μg/mL) for *S. epidermidis*, respectively. The MIC_90_ values were 12.5 μM (approximately 3.67 μg/mL) for MSSA, MRSA, *S. epidermidis*, and *E. faecium*, but increased to 25 μM (approximately 7.34 μg/mL) for *E. faecalis*. To make a standardized comparison and include a clinically relevant strain, we chose ATCC 29213 (MSSA), USA300 (MRSA), and YUSA145 (a laboratory-stored clinical MRSA strain) (20). The automated bacterial growth curves further confirmed that at 1× MIC concentration (12.5 μM), PFM01 effectively inhibited the planktonic growth of these representative *S. aureus* strains (Fig. 1b through d). The results of the time-kill assay showed that, for the stationary-phase cultures of ATCC 29213 and YUSA145, PFM01 at a concentration of 200 μM (approximately 58.68 μg/mL; 16× MIC) reduced the bacterial load within the first 24 h of treatment, and by 96 h had reduced the *S. aureus* load by 3–4 log_10_ CFU/mL. In contrast, Van (32 μg/mL) exhibited limited antibacterial activity against the *S. aureus* stationary-phase cells under the same conditions (Fig. 1e and f). Furthermore, PFM01 at a concentration of 50 μM (approximately 14.67 μg/mL; 4× MIC) exhibited rapid bactericidal activity against YUSA145 cells in the logarithmic growth phase, reducing the viable cell count by approximately 3.5 log_10_ CFU/mL within 6 h; at this point, its bactericidal activity was more pronounced than that of Van (16 μg/mL) (Fig. 1g).

**TABLE 1:** MIC of PFM01 against Gram-positive bacteria^*a*^

Strains	PFM01 MIC distributions (μM)				
	6.25	12.5	25	MIC_50_	MIC_90_
*MSSA* (*n* = 41)	4 (9.76%)	36 (87.80%)	1 (2.44%)	12.5	12.5
*MRSA* (*n* = 39)	3 (7.69%)	35 (89.74%)	1 (2.56%)	12.5	12.5
*S. epidermidis* (*n* = 22)	18 (81.82%)	4 (18.18%)	0	6.25	12.5
*E. faecium* (*n* = 19)	0	18 (94.74%)	1 (5.26%)	12.5	12.5
*E. faecalis* (*n* = 21)	2 (9.52%)	11 (52.38%)	8 (38.10%)	12.5	25

^*a*^ MIC, minimum inhibitory concentration; MIC_50_/MIC_90_, the MIC values for 50% or 90% of bacterial growth inhibition.

**Fig 2:**
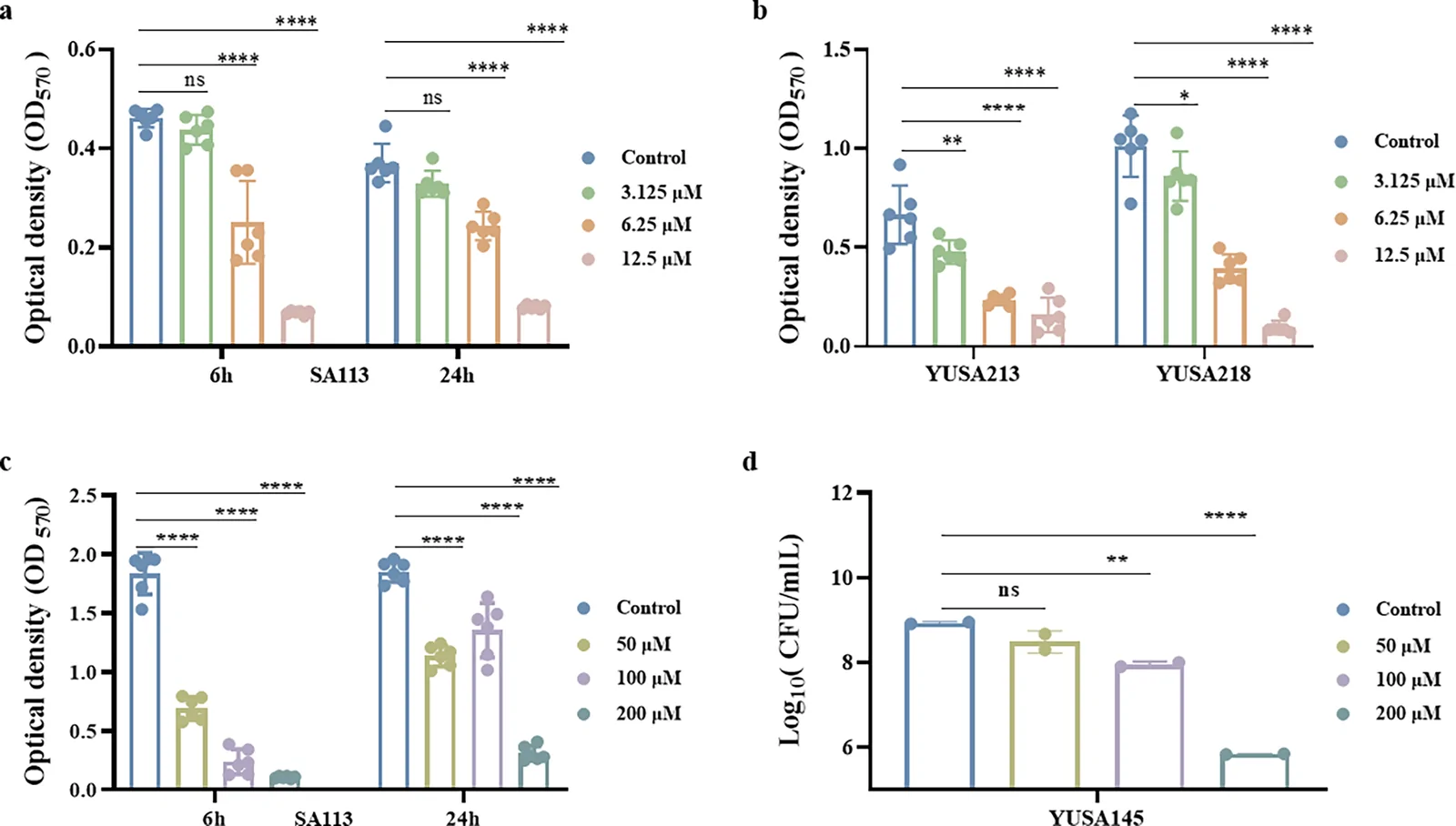
Inhibition of biofilm formation and eradication of mature biofilms by PFM01 against *S. aureus*. (**a**) Inhibition of biofilm formation of SA113 by PFM01 at 6 and 24 h. (**b**) Inhibition of biofilm formation in YUSA213 and YUSA218 by PFM01 at 24 h. (**c**) The eradication effect of PFM01 on mature biofilms of SA113 at 6 and 24 h. (**d**) The eradication effect of PFM01 on mature biofilms of YUSA145. *n* = 3, and data are presented as mean ± SD. *P < 0.05; **P < 0.01; ***P < 0.001; ****P < 0.0001; (ANOVA).

We then evaluated the anti-biofilm effects of PFM01 and its ability to eradicate mature biofilms. At a concentration of 1/2× MIC (6.25 μM), PFM01 inhibited biofilm formation by SA113 (MSSA) at both 6 and 24 h (Fig. 2a). At a concentration of 1/4× MIC (3.125 μM), PFM01 similarly inhibited biofilm formation by two clinical MRSA strains (YUSA213 and YUSA218) (Fig. 2b). Furthermore, PFM01 at a concentration of 4× MIC (50 μM) reduced the formation of mature biofilms by SA113 within 6 h and retained its inhibitory effect at 24 h (Fig. 2c), while PFM01 at a concentration of 8× MIC (100 μM) also had a clearing effect on the mature biofilm of the MRSA strain YUSA145 (Fig. 2d).

### *In vitro* biosafety assessment of PFM01

**Fig 3:**
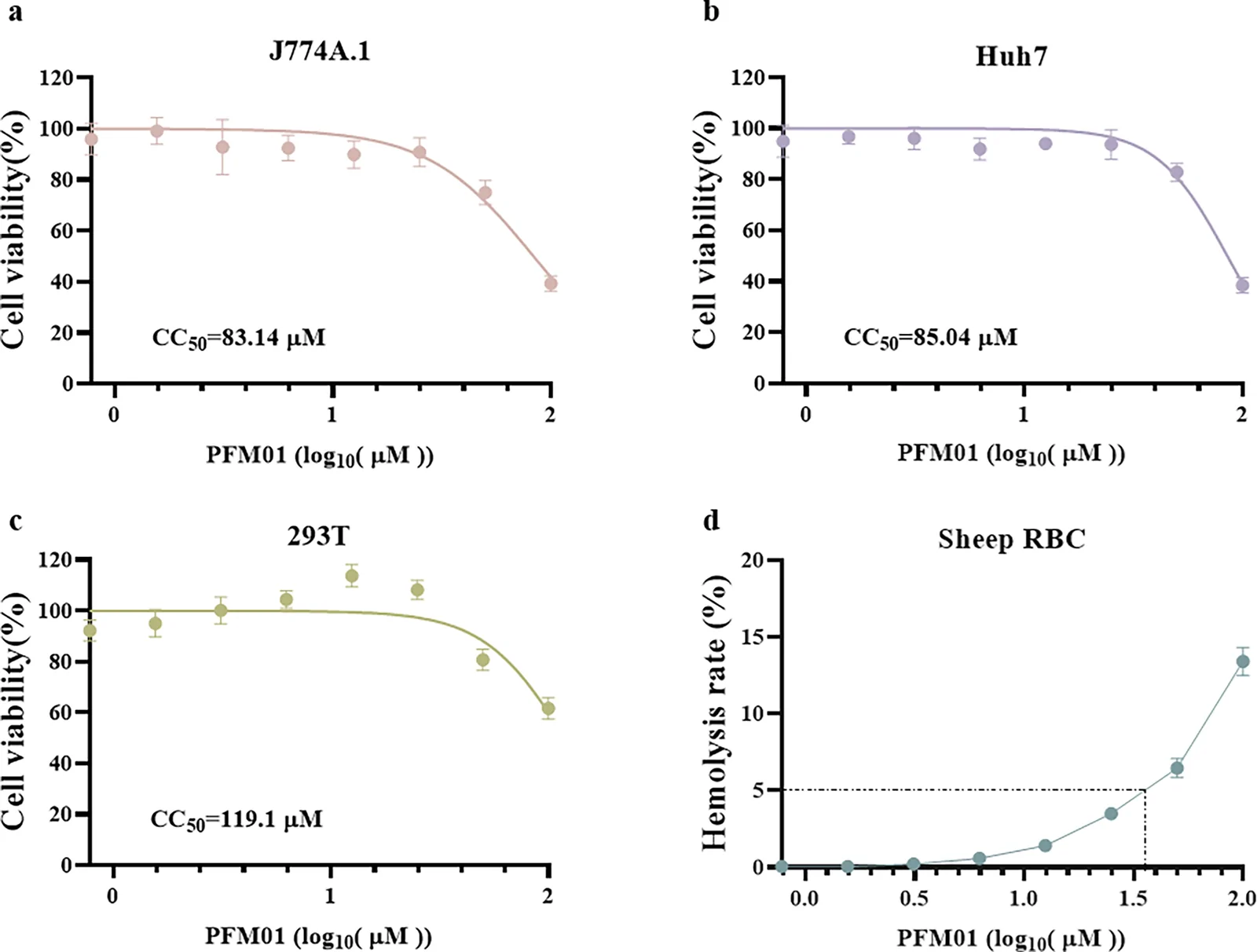
*In vitro* cytotoxicity and hemolytic activity of PFM01. (**a–c**) The cytotoxicity of PFM01 to J774A.1 cell (**a**), Huh7 cell (**b**), and 293T cell (**c**) was measured by the CCK-8 assay. (**d**) The hemolytic activity of PFM01 on sheep erythrocytes. Triton X-100 serves as the positive control and PBS serves as the negative control. *n* = 3, and data are presented as mean ± SD.

Cytotoxicity of PFM01 was assessed using the CCK-8 assay on mouse monocyte macrophage J774A.1, human hepatoma Huh7, and human embryonic kidney 293T cell lines. The results indicated that PFM01 displayed a favorable safety profile within its effective antibacterial concentration range (6.25–25 μM). The half-maximal cytotoxic concentrations (CC_50_) are determined to be 83.14 μM (approximately 24.39 μg/mL) for J774A.1 (Fig. 3a), 85.04 μM (approximately 24.95 μg/mL) for Huh7 (Fig. 3b), and 119.1 μM (approximately 34.94 μg/mL) for 293T cells (Fig. 3c), values substantially higher than its antibacterial MICs. To provide a standardized assessment of its therapeutic window, we used the lowest CC_50_ value (83.14 μM for J774A.1 cells) as a conservative estimate and calculated the selectivity index (SI = CC_50_/MIC). As shown in Table 2, the SI values ranged from 3.3 to 13.3 depending on the strain and the MIC endpoint used, indicating that selectivity is strain-dependent.

**TABLE 2:** Selectivity indices (SIs) of PFM01 against different Gram-positive bacterial strains^*a*^

Strain	MIC_50_ (μM)	MIC_90_ (μM)	SI (based on MIC_50_)	SI (based on MIC_90_)
MSSA (*n* = 41)	12.5	12.5	6.7	6.7
MRSA (*n* = 39)	12.5	12.5	6.7	6.7
*S. epidermidis* (*n* = 22)	6.25	12.5	13.3	6.7
*E. faecium* (*n* = 19)	12.5	12.5	6.7	6.7
*E. faecalis* (*n* = 21)	12.5	25	6.7	3.3

^*a*^ SI was calculated using the lowest CC_50_ value (83.14 μM against J774A.1 cells) to provide a conservative estimate. Values are rounded to one decimal place. SI = CC_50_/ MIC.

Furthermore, the hemolytic activity of PFM01 was tested against sheep red blood cells (RBCs), using 25% Triton X-100 as a 100% hemolysis control and PBS as a negative control. No significant hemolysis was observed at concentrations corresponding to its effective antibacterial range (Fig. 3d).

### The disrupted membrane of *S. aureus* by PFM01

**Fig 4:**
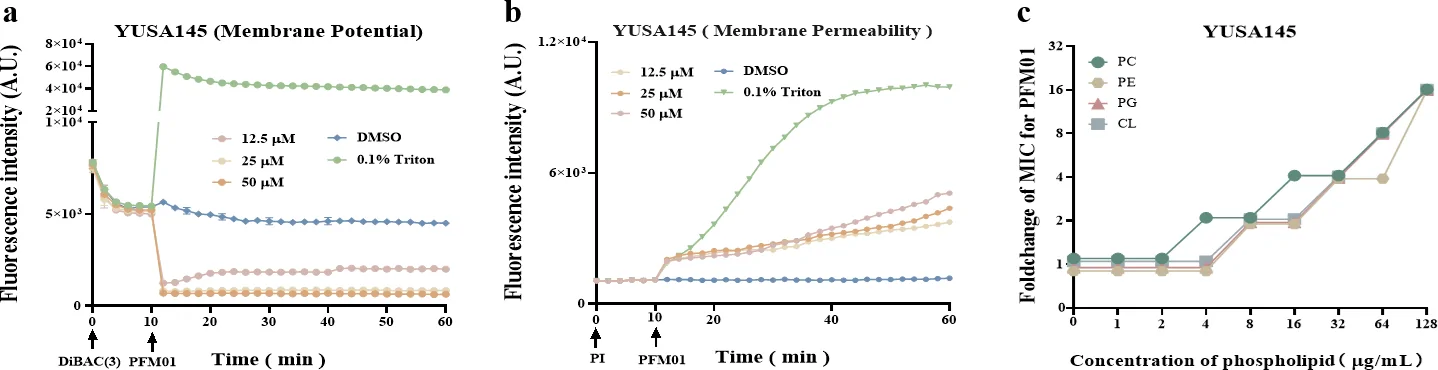
Injurious effects of PFM01 on the bacterial cell membrane. to evaluate the effects of PFM01 at different concentrations on *S. aureus* YUSA145 in terms of membrane potential (DiBAC_4_(3) staining) (**a**) and membrane permeability (PI staining) (**b**). The effect of exogenous phospholipids (including phosphatoglycerol [PG], phosphatidylcholine [PC], phosphatidylethanolamine [PE], and cardiolipin [CL]) on the antibacterial activity of PFM01, as determined by the checkerboard assay (**c**).

Since cell membranes are a common target for many antimicrobials, we checked out how PFM01 directly affects the cell membrane integrity of YUSA145. Propidium iodide (PI) was used to quantify changes in membrane permeability in the *S. aureus* strain YUSA145, while DiBAC_4_(3) to monitor changes in membrane potential in the *S. aureus* strain YUSA145. Upon exposure to PFM01 at a concentration of 12.5 μM, a significant increase in PI uptake and DiBAC_4_(3) fluorescence intensity was detected, indicating enhanced membrane permeability and membrane depolarization; these effects were concentration-dependent (Fig. 4a and b).

Phospholipids are key components of the *S. aureus* cell membrane. To investigate the potential antibacterial mechanism of PFM01, we further examined the effect of exogenous phospholipids on the antibacterial activity of PFM01. Checkerboard assay results showed that the antibacterial activity of PFM01 against *S. aureus* YUSA145 was reduced in the presence of phosphatidylglycerol (PG), phosphatidylcholine (PC), phosphatidylethanolamine (PE), or cardiolipin (CL); when 128 μg/mL of these phospholipids was added to the culture medium, the antibacterial activity of PFM01 against YUSA145 decreased 16-fold (Fig. 4c).

### Integrated proteomic analysis reveals membrane-centric stress and adaptive responses of *S. aureus* to PFM01 exposure

**Fig 5:**
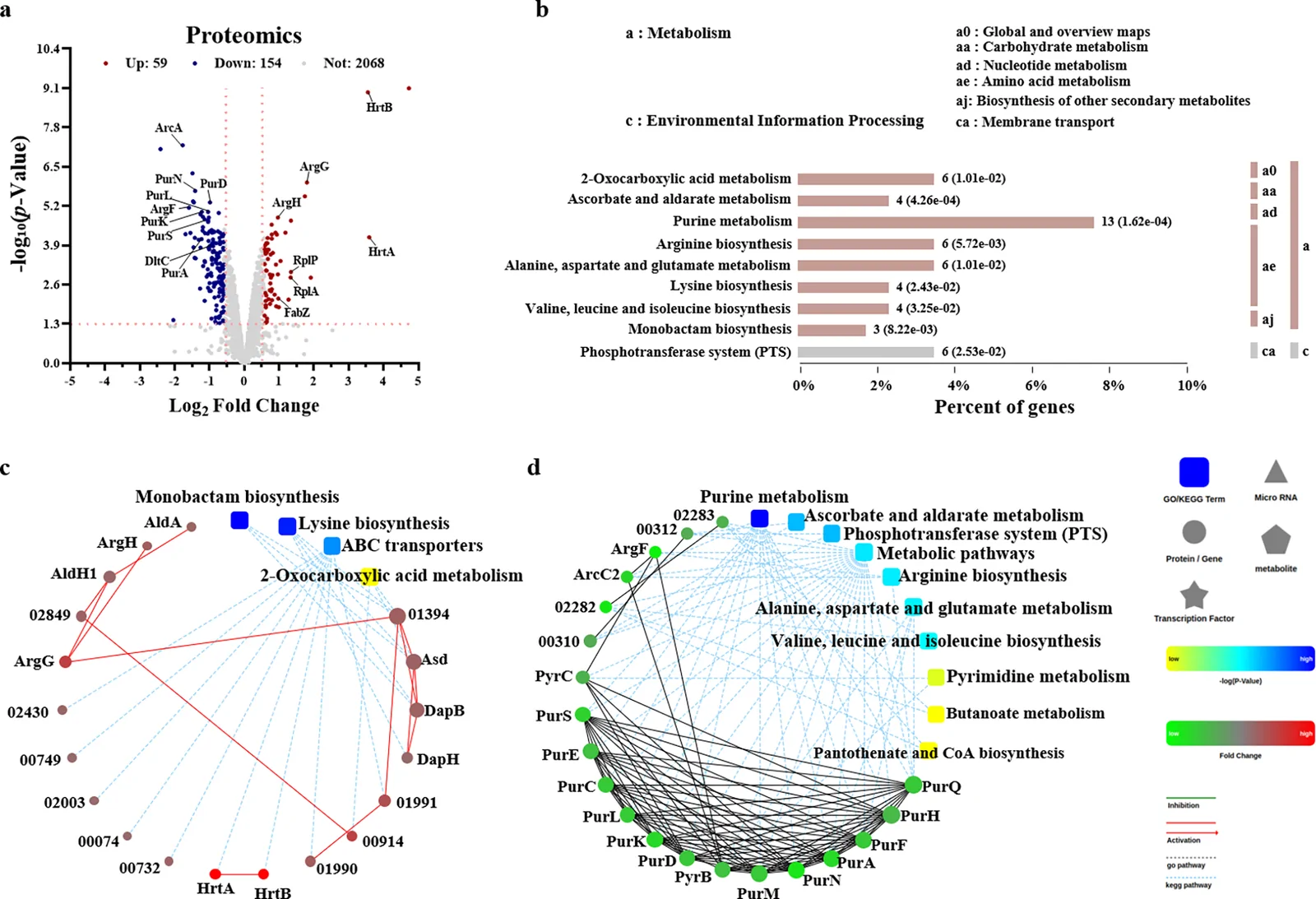
Global proteomic response of *S. aureus* SA113 to PFM01. (**a**) Volcano plot of differentially expressed proteins, with 59 upregulated proteins (red) and 154 downregulated proteins (blue). (**b–d**) Bioinformatic analyses performed using the OmicsBean online platform: classification of enriched KEGG pathways (**b**) and protein-protein interaction (PPI) networks of upregulated (**c**) and downregulated (**d**) proteins.

To investigate the molecular response mechanisms of *S. aureus* SA113 in response to PFM01, a global proteomics analysis was conducted in this study. Following treatment of the strain with 12.5 μM PFM01 for 4 h, total cellular proteins were extracted and analyzed by liquid chromatography–tandem mass spectrometry (LC–MS/MS) for qualitative and quantitative detection, resulting in the identification of 2,281 proteins. Using a screening criterion of |log_2_ fold change| > 0.587 and *P* ≤ 0.05, a total of 213 differentially expressed proteins were identified through intergroup comparison, comprising 59 upregulated proteins and 154 downregulated proteins (Fig. 5a; Table S1). KEGG pathway enrichment analysis based on the OmicsBean platform revealed that purine metabolism was the most significantly differentially expressed pathway following treatment with PFM01; furthermore, several core metabolic processes, including amino acid biosynthesis, organic acid metabolism, and the phosphotransferasesystem (PTS), were also significantly enriched (Fig. 5b).

Further analysis of the upregulated proteins revealed that PFM01 triggers a synergistic stress response in the bacteria, with proteins associated with membrane stress and functional adaptation showing marked compensatory upregulation. Among these, the HrtA/HrtB ABC transport system, which mediates responses to heme and oxidative stress, was significantly induced (HrtA: log_2_ FC = 3.59; HrtB: log_2_ FC = 3.55). Concurrently, the expression of FabZ, a key enzyme in fatty acid elongation (log_2_ FC = 0.979), and CorA, a major magnesium ion transporter (log_2_ FC = 0.869), was upregulated, suggesting that the bacterium attempts to counteract drug stress by remodeling membrane lipid homeostasis and ion balance (Fig. 5c).

In contrast, PFM01 significantly downregulated a series of key proteins involved in maintaining cell membrane structure and cell wall biosynthesis, including AcpP (acyl carrier protein), a core protein in fatty acid synthesis (log_2_ FC = −1.14), the Lipoteichoic acid synthase LtaS (log_2_ FC = −0.875), and DltC (D-alanine carrier protein, log_2_ FC = −1), which is responsible for the modification of membrane surface charge. Notably, almost all key enzymes in the *de novo* purine synthesis pathway were inhibited, including a series of rate-limiting and structural enzymes, such as PurN, PurA, PurK, PurS, PurL, PurC, PurQ, PurF, PurM, PurD, PurE, and PurH (log_2_ FC ranging from −1.41 to −0.789). Purine metabolism forms the cornerstone of bacterial synthesis of DNA, RNA, and energy molecules such as ATP; the overall downregulation of the aforementioned proteins indicates that PFM01 severely disrupts the synthesis of genetic material and energy supply within the bacterial cells. Concurrently, proteins associated with core intracellular biosynthetic pathways, such as amino acid metabolism, were also widely inhibited (Fig. 5d).

### Whole-genome sequencing of PFM01-induced resistant *S. aureus*

To further investigate the potential mechanisms underlying *S. aureus* resistance to PFM01, this study used the wild-type susceptible strain SA113 as the target strain and successfully obtained PFM01-induced strain through continuous induction with gradually increasing drug concentrations *in vitro*. Subsequently, whole-genome sequencing was employed to perform genome-wide comparative analysis between the parental susceptible strain SA113 and the corresponding PFM01-induced strain, with the aim of identifying key gene mutations associated with the resistance phenotype. The results identified a total of six mutation sites, of which five were nonsynonymous and one was synonymous (Table 3). Among these genes harboring potential resistance-associated mutations, the MprF protein (phosphatidylglycerol lysyltransferase) carries an R186X nonsense mutation (21). As a key membrane-modifying protein in *S. aureus*, MprF is closely associated with cell membrane stability and resistance to antimicrobial peptides. This mutation is highly likely to constitute the key molecular basis for resistance to PFM01. Furthermore, the ClpX protein (ATP-binding subunit of the ATP-dependent Clp protease) carries an A326V missense mutation (22). This protein is involved in bacterial protein quality control and stress responses; this mutation may enhance bacterial tolerance to PFM01 exposure. The aforementioned mutations may confer PFM01 resistance in *S. aureus* by affecting cell membrane structural modification and protein homeostasis regulation.

**TABLE 3:** Genetic mutations in PFM01-induced resistant *S. aureus* SA113

Gene_ID	Nucleotide mutation	Amino acid mutation	Mutation type	Subject description
SA113_GM000259	G62A	G21D	Nonsynonymous	RNase adapter RapZ
SA113_GM000726	C976T	D326N	Nonsynonymous	Threonine aldolase, partial or low specificity L-threonine aldolase or threonine aldolase [MupR]
SA113_GM000771	A556T	R186X	Nonsynonymous	Bifunctional lysylphosphatidylglycerol flippase/synthetase MprF
SA113_GM001901	T528C	N176N	Synonymous	Staphylocoagulase
SA113_GM002146	G977A	A326V	Nonsynonymous	ATP-dependent Clp protease ATP-binding subunit ClpX
SA113_GM002380	G1900A	P406S	Nonsynonymous	Phage terminase, large subunit subunit or PBSX family phage terminase large subunit

In addition, we generated *mprF* and *clpX* single-gene knockout strains (Δ*mprF* and Δ*clpX*) from the susceptible strain SA113. Analysis of antimicrobial activity and bacterial growth curves showed that the MIC of PFM01 against Δ*mprF* and Δ*clpX* did not change significantly. Under PFM01 exposure, the growth trend of Δ*mprF* was comparable to that of the parental strain, whereas at 12.5 µM PFM01, the parental strain exhibited no growth while the Δ*clpX* strain still showed detectable growth. At 25 µM PFM01, growth of the Δ*clpX* strain was also completely inhibited (Fig. S1; Table S2). These results suggest that *mprF* and *clpX* may only function in the acquisition of PFM01 resistance during induction, rather than directly mediating the intrinsic susceptibility of *S. aureus* to PFM01.

### Identification of a potential direct target of PFM01 via DARTS assay

**Fig 6:**
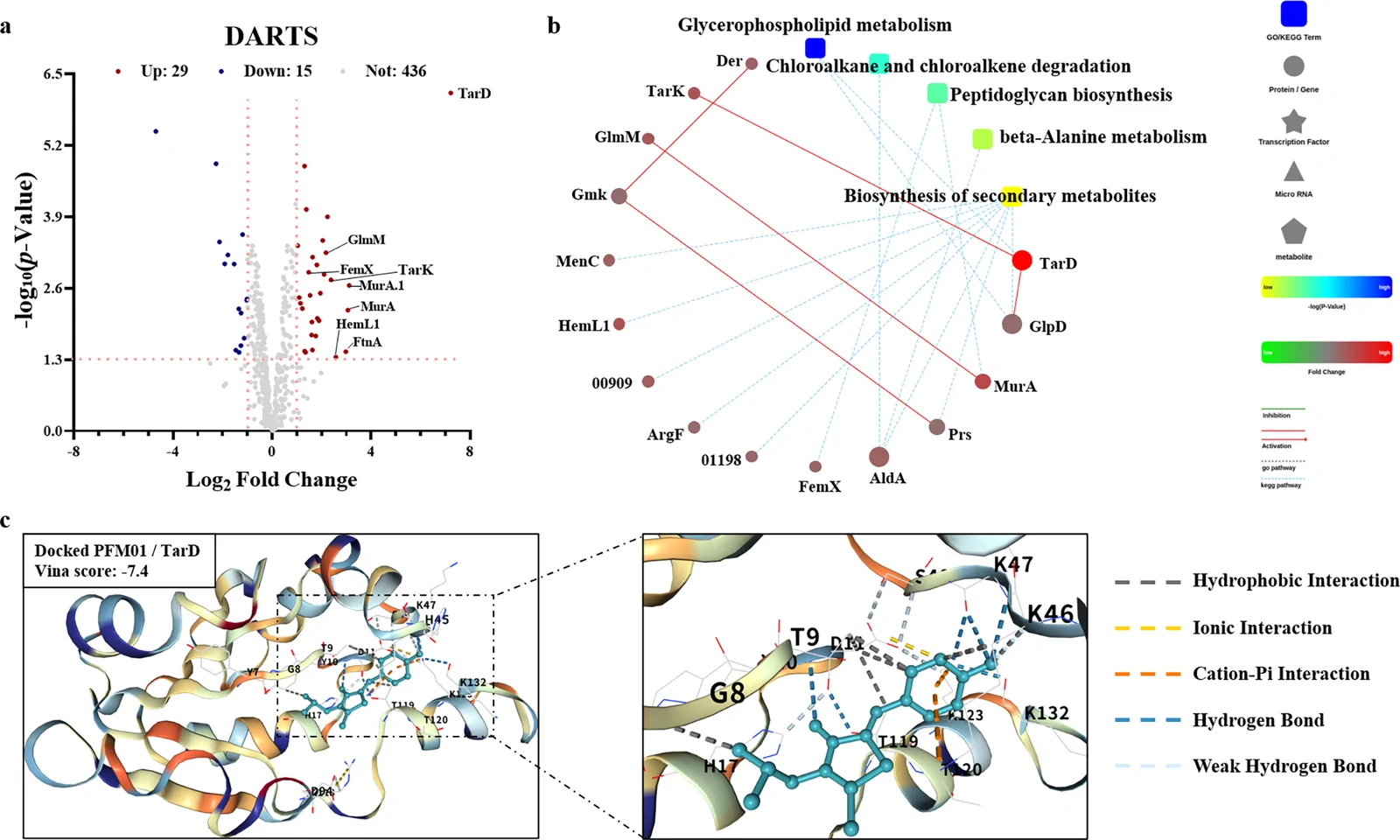
Identification of the potential antibacterial target of PFM01 using the DARTS assay. (**a**) Volcano plot of differentially expressed proteins detected by LC-MS/MS, with 29 upregulated proteins (red) and 15 downregulated proteins (blue). (**b**) Protein-protein interaction (PPI) network of the stabilized (upregulated) proteins. (**c**) Molecular docking model of PFM01 with the candidate target protein TarD, showing a Vina score of −7.4 kcal/mol (simulated using the CB-Dock two online platform).

Having preliminarily characterized the proteomic expression profile of *S. aureus* SA113 under PFM01 stress and identified drug resistance-associated mutation sites, this study further focused on screening the direct targets of PFM01 and elucidating its regulatory mechanisms underlying bacterial growth and core metabolic processes. To this end, we performed DARTS assays. Protein identification was conducted using LC-MS/MS, and data analysis was performed on the OmicsBean platform. A total of 480 proteins were identified. Based on the screening criteria (log_2_ fold change >1 and *P*-value ≤ 0.05), 29 proteins that were stabilized by PFM01 were identified (Table S3). Using the OmicsBean platform, we created a data volcano plot (Fig. 6a) and performed KEGG pathway enrichment analysis for the proteins with stable level in PFM01; the results showed that glycerophospholipid metabolism was a significantly enriched category. At the same time, we built a protein-protein interaction (PPI) network for these stabilized proteins. (Fig. 6b).

Among the upregulated proteins, TarD exhibited the most significant change (log_2_ FC = 7.22). TarD catalyzes the transfer of a cytidine 5'-phosphate (CTP) group to sn-glycero-3-phosphate, generating activated glycero-3-phosphate (23). This activated glycero-3-phosphate is then incorporated into the linker unit via TarB and TarF, thereby participating in the biosynthesis of teichoic acid in the cell wall. To elucidate the interaction mechanism between PFM01 and TarD, molecular docking simulations were performed using the CB-Dock two platform. The results showed that PFM01 binds to the active pocket of TarD through multiple non-covalent interactions, including six conventional hydrogen bonds, five weak hydrogen bonds, two cation-π interactions, and one ionic interaction, involving 35 amino acid residues in total. The predicted binding affinity (Vina score) was −7.4 kcal/mol (Fig. 6c).

Based on the DARTS screening results, TarD was identified as a potential high-affinity direct target of PFM01. Molecular docking simulations further supported the formation of a stable complex between PFM01 and the active site of TarD. However, the direct regulatory effect of PFM01 on TarD enzymatic activity and its detailed antibacterial mechanism warrant further experimental validation.

## DISCUSSION

The global spread of MRSA has created an urgent need for novel antimicrobial agents (24). Drug repurposing has proven to be an effective and cost-efficient strategy for antimicrobial development (25). For instance, derazantinib, initially developed as an FGFR 1-3 inhibitor for intrahepatic cholangiocarcinoma, has recently been shown to effectively inhibit *S. aureus* growth and attenuate biofilm formation by binding to membrane phospholipids and disrupting cell membrane integrity (14). Our findings suggest that PFM01 may represent another promising example of this approach, providing a feasible strategy for the discovery of new antimicrobial agents. In the present study, PFM01, an N-alkylated derivative originally developed as an MRE11 inhibitor, was identified as an antibacterial candidate, further supporting the potential of drug repurposing approaches for the discovery of novel antimicrobial agents.

Our findings provide the first evidence that PFM01 exhibits broad-spectrum antimicrobial activity against Gram-positive bacteria, including MSSA, MRSA, and Enterococci. Time-kill assays further demonstrated that stronger bactericidal activity was exerted by PFM01 than Van against stationary-phase and adherent bacterial populations. The ability of PFM01 to retain antibacterial activity against metabolically inactive bacterial populations is particularly noteworthy, as such populations are frequently associated with persistent infections and reduced antibiotic susceptibility (26). During experiments performed with log-phase bacteria, regrowth was observed following PFM01 treatment but not Van treatment, suggesting that a relatively narrow bactericidal window or slower bactericidal kinetics may be associated with PFM01 under these conditions (27). Further pharmacokinetic and pharmacodynamic investigations will be required to characterize PFM01 exposure profiles, stability, and optimized dosing strategies under physiological conditions.

Biofilm formation represents a major challenge in the treatment of bacterial infections, as biofilm-associated cells frequently exhibit enhanced tolerance to conventional antibiotics (28). In this study, PFM01 was shown to effectively inhibit biofilm formation and eradicate preformed mature biofilms in a dose-dependent manner. This activity may be associated with its ability to retain antibacterial activity against slow-growing bacterial populations, as demonstrated by its sustained killing effect against stationary-phase *S. aureus* cells. Furthermore, proteomic analysis revealed significant alterations in proteins involved in cell envelope maintenance and stress adaptation, including decreased expression of DltC (29) and LtaS (30), which are involved in teichoic acid modification and lipoteichoic acid biosynthesis, respectively. Given the important roles of these envelope-associated pathways in bacterial adhesion and biofilm maturation (31), their dysregulation may contribute to the impaired biofilm development observed following PFM01 exposure. Additional changes in membrane lipid metabolism-associated proteins, such as AcpP (32), and stress-response proteins such as CopZ (33), further suggest that PFM01 induces broad cellular alterations beyond growth inhibition. Future imaging-based studies will help clarify the spatial distribution and viability of PFM01-treated bacterial populations within mature biofilms.

The cell envelope of Gram-positive bacteria contains abundant negatively charged components, including teichoic acids, lipoteichoic acids, and anionic phospholipids, which represent important targets for antimicrobial intervention (34). For example, daptomycin exerts bactericidal activity by interacting with phosphatidylglycerol and disrupting membrane homeostasis (35). In the present study, several lines of evidence suggested that PFM01 exerts a membrane-associated antibacterial effect. The antibacterial activity of PFM01 was attenuated by exogenous phospholipid supplementation, and PI and DiBAC_4_(3) staining further demonstrated that PFM01 impaired membrane permeability and membrane potential in *S. aureus*. Notably, the membrane-targeting mechanism of PFM01 is consistent with the general antibacterial strategies previously reported by our laboratory. Our team’s research has confirmed that repurposed drugs, such as AMXT-1501 (20) and fingolimod (36) exert antibacterial effects by acting on bacterial membranes, further validating the universality of this membrane-targeting strategy.

Proteomic analysis further revealed that PFM01 induced significant alterations in proteins associated with cell envelope maintenance and membrane metabolism. The downregulation of DltC and LtaS, together with changes in AcpP and FabZ expression, suggested that PFM01 might disrupt bacterial envelope homeostasis. Furthermore, TarD (37), a key enzyme involved in wall teichoic acid biosynthesis, was identified as a potential interacting protein of PFM01 by DARTS analysis, and molecular docking predicted a possible interaction between PFM01 and TarD. Mutations identified in *mprF* (38) and *clpX* (39) from PFM01-adapted strains did not significantly alter PFM01 susceptibility, suggesting that these changes may represent adaptive responses rather than primary resistance mechanisms (40–42). Together, these findings suggest that PFM01 may impair *S. aureus* cell envelope integrity through modulation of WTA-associated pathways, subsequently contributing to membrane perturbation. However, whether membrane perturbation represents a direct effect of PFM01 or a secondary consequence of TarD-associated envelope disruption requires further investigation.

This study has several limitations that warrant further investigation. First, the antibacterial activity and biosafety of PFM01 require validation in relevant *in vivo* infection models. Second, although TarD was identified as a potential interacting protein of PFM01, additional biochemical and biophysical studies are needed to confirm its role in PFM01-mediated antibacterial activity. Third, long-term evolution experiments and systematic structure-activity relationship studies will be required to evaluate the potential for resistance development and optimize the antibacterial properties of PFM01 derivatives. Fourth, while our current data support a membrane-disrupting mechanism, direct visualization of PFM01-induced morphological changes to the bacterial membrane via transmission electron microscopy (TEM) would provide more definitive evidence. Fifth, the biofilm experiments in this study were performed using crystal violet staining as a straightforward method for initial quantification. Future studies employing SYTO9/PI staining and confocal microscopy are needed to further explore the anti-biofilm activity of PFM01. Finally, although PFM01 shows significant antibacterial activity against Gram-positive bacteria, its efficacy against Gram-negative bacteria, particularly drug-resistant strains, remains unevaluated. Future studies should systematically assess its activity against Gram-negative bacteria, explore its synergistic potential with polymyxin B, and verify its efficacy against polymyxin B-resistant strains to expand its clinical applicability.

### Conclusion

In conclusion, this study identified PFM01 as a promising repurposed antibacterial candidate against Gram-positive pathogens, particularly multidrug-resistant *S. aureus*. PFM01 exhibited potent bactericidal activity against both planktonic and biofilm-associated bacterial populations, while showing favorable *in vitro* biosafety profiles. Mechanistically, PFM01 was shown to induce membrane-associated damage and perturb cell envelope homeostasis, with TarD identified as a potential interacting protein involved in its antibacterial activity. Whole-genome analysis further suggested that mutations in *mprF* and *clpX* represent adaptive responses rather than primary resistance determinants. Collectively, these findings highlight PFM01 as a potential lead compound for further preclinical development against drug-resistant *S. aureus* infections.

## MATERIALS AND METHODS

### Bacterial strains and growth conditions

All chemicals and reagents were obtained from commercial sources. PFM01 was sourced from the Chemicals Screening Library (MedChemExpress, Shanghai, China). The library comprised 1,000 compounds with diverse structures, from which PFM01 was identified through a two-step screening process: initial screening at two fixed concentrations (12.5 and 50 μM) against *S. aureus* strains USA300 and SA113 using the broth microdilution method, followed by full MIC determination for hit compounds. Antibiotic controls, vancomycin (Van), alongside experimental reagents including phosphate-buffered saline (PBS), Propidium Iodide (PI), DiBAC_4_(3), and the cell counting kit-8 (CCK-8), were also procured from MedChemExpress. Bacterial culture media, cation-adjusted Mueller−Hinton broth (CAMHB) and tryptic soy broth (TSB), were acquired from Huankai Microbial (Guangzhou, China). The bacterial strains used in this study included *S. aureus* ATCC 29213 (MSSA reference strain), SA113 (MSSA laboratory strain), YUSA145 (clinical MRSA isolate), YUSA213 (clinical MRSA isolate), YUSA218 (clinical MRSA isolate), USA300 (MRSA reference strain), *S. epidermidis* (clinical isolate), *E. faecium* (clinical isolate), and *E. faecalis* (clinical isolate). The selection of strains is based on their relevance as standard reference strains (ATCC 29213, USA300) for comparative research studies, or as clinically common isolates (YUSA145, YUSA213, YUSA218, SA113, and enterococci) for comparison with antibiotics used in clinical practice. The specific strains used in each experiment will be indicated in the corresponding Methods section below.

### Determination of minimum inhibitory concentration (MIC)

MIC of PFM01 against each bacterial strain was determined using the broth microdilution method in cation-adjusted Mueller-Hinton broth (CAMHB) following the Clinical and Laboratory Standards Institute (CLSI) guidelines. Overnight cultures of the strains were diluted 1:1,000 in CAMHB and added to a 96-well round-bottom microplate. A series of twofold serial dilutions of PFM01 were prepared from a 10-mM stock solution, yielding final concentrations ranging from 200 to 1.56 μM (with a final DMSO concentration of 2% (v/v) at the highest concentration), and an equal volume of each dilution was added to the corresponding wells. Control wells received an equal volume of DMSO, the solvent used to dissolve PFM01. After 24 h of incubation at 37°C, the optical density at 595 nm (OD_595_) was measured, and the results were confirmed by visual inspection. The MIC was defined as the lowest concentration of PFM01 that completely inhibited visible bacterial growth. The MIC_50_ and MIC_90_ values are defined as the MICs that inhibit 50% and 90% of the tested isolates, respectively, and are determined based on the distribution of individual MIC values of all the tested isolates.

### Determination of bacterial growth curves

Overnight cultures of *S. aureus* strains ATCC 29213, YUSA145, and USA300 were diluted 1:100 in fresh TSB and added to specialized 100-well Honeycomb plates (with three replicates), with an initial concentration of approximately 5 × 10^6^ CFU/mL. PFM01 was diluted to final concentrations of 2× MIC (25 μM, with a final DMSO concentration of 0.25% [v/v]), 1× MIC, 1/2× MIC, and 1/4× MIC, and equal volumes were added to the wells. The plates were placed in a growth curve analyzer (Bioscreen, Finland), and the OD_600_ absorbance was measured every hour for 24h. Growth curves were plotted based on the measurements.

### Time-Dependent killing assay

Time-kill curve assays were performed to evaluate the bactericidal activity of PFM01 against both stationary-phase and log-phase *S. aureus* cells. For assays against stationary-phase bacteria, strains YUSA145 and ATCC 29213 were first cultured to the stationary phase. The cultures were then aliquoted into equal volumes and treated with PFM01 at a final concentration of 200 µM (16× MIC; with a final DMSO concentration of 2% [v/v]), with Van (32 µg/mL) and an equivalent volume of DMSO serving as the positive and vehicle controls, respectively. Bacterial viability was determined by CFU counts on Tryptic Soy Agar (TSA) plates at 24-h intervals over a 120-h period. For assays against log-phase bacteria, strain YUSA145 was used. Bacterial suspensions in the logarithmic growth phase were adjusted to approximately 1 × 10^6^ CFU/mL in TSB and treated with PFM01 at a final concentration of 50 µM (4× MIC, with a final DMSO concentration of 0.5% [v/v]). Van (16 µg/mL) and DMSO were used as the positive and vehicle controls, respectively. All cultures were incubated with shaking at 37°C, and samples were collected at 0, 3, 6, and 24 h for serial dilution, plating, and subsequent CFU enumeration. The concentrations of PFM01 used in these experiments (200 µM for stationary-phase cells and 50 µM for logarithmic-phase cells) were selected based on preliminary dose screening experiments to achieve optimal bactericidal activity.

### Biofilm formation and eradication assays

The anti-biofilm activity of PFM01 was evaluated through a 96-well plate model using crystal violet staining, encompassing two complementary strategies: inhibition of biofilm formation and eradication of pre-formed biofilms. For the biofilm inhibition assay, overnight cultures of specific strains were used. *S. aureus* strain SA113 was employed to assess inhibition after both 6 and 24 h of treatment. Strains YUSA213 and YUSA218 were used specifically for the 24-h inhibition assessment. Each bacterial culture was diluted in TSBG medium (Tryptic Soy Broth supplemented with 1% glucose) containing serially diluted PFM01 at final concentrations of 3.125, 6.25, and 12.5 μM (corresponding to 1/4×, 1/2×, and 1× MIC, respectively; the final DMSO concentration at 12.5 μM was 0.125%, v/v) and incubated statically at 37°C for the designated durations to allow biofilm development in the presence of the compound. For the biofilm eradication assay, mature biofilms were first established. Strain SA113 was used to evaluate eradication after 6 and 24 h of PFM01 exposure, while strain YUSA145 was used for the 24-h eradication assessment. For SA113 and YUSA145, the tested concentrations of PFM01 were 50, 100, and 200 μM (corresponding to 4×, 8×, and 16× MIC, respectively; the final DMSO concentration at 200 μM was 2%, v/v). Biofilms were formed by incubating a 1:200 dilution of an overnight culture in TSBG (without drug) statically for 24 h. The mature biofilms were then treated with fresh TSBG medium containing PFM01 for an additional 6 or 24h.

Following the respective incubations for both assays, non-adherent cells and medium were removed. The remaining biofilm was washed twice with PBS, air-dried, fixed with methanol for 15 min, and allowed to dry again. The fixed biofilms were then stained with 0.1% crystal violet solution for 15 min. After staining, unbound dye was removed by thorough washing with water. The plates were dried, and the biofilm biomass was quantified by directly measuring the optical density at 570 nm.

### Assessment of hemolytic activity and cytotoxicity

The hemolytic potential of PFM01 against mammalian cells was evaluated using a sheep erythrocyte model. Briefly, fresh sheep red blood cells were washed with PBS and resuspended as a 4% (v/v) solution in PBS. Aliquots (100 µL) of this suspension were added to a 96-well plate and mixed with an equal volume of serially diluted PFM01. A solution of 25% Triton X-100 served as the positive control for complete hemolysis. Following incubation at 37°C for 1 h, the plate was centrifuged at 1,000 × *g* for 5 min. Subsequently, 100 µL of the supernatant from each well was transferred to a new plate, and the absorbance at 540 nm was measured to quantify the released hemoglobin.

Cytotoxicity was determined using the CCK-8 assay. Three mammalian cell lines were used: mouse mononuclear macrophage J774A.1, human hepatocellular carcinoma Huh7, and human embryonic kidney 293T cells. Cells were initially cultured overnight in complete DMEM medium at 37°C in a 5% CO_2_ atmosphere. The cell suspension was then adjusted and seeded into a 96-well plate at a density of 6,000 cells per well. After a 24-h adherence period, the cells were treated with various concentrations of PFM01 (ranging from 200 to 1.56 μM) for an additional 24h. Following treatment, the CCK-8 reagent was added to each well, and the plates were incubated for 1 h at 37°C. The absorbance at 450 nm was recorded using a microplate reader, and cell viability was expressed as a percentage relative to the untreated control group.

### Bacterial membrane permeability assay

Membrane permeability was assessed using the fluorescent dye PI, which enters cells with compromised membranes. *S. aureus* strain YUSA145 was cultured to the exponential-growth phase, harvested by centrifugation, washed with PBS, and resuspended to an optical density at 600 nm (OD_600_) of 0.5. The bacterial suspension was incubated with PI at a final concentration of 1 μM in the dark for 20 min. Subsequently, aliquots were treated with PFM01 at final concentrations of 12.5, 25, and 50 μM (with a final DMSO concentration of 0.5%, v/v). A suspension treated with 0.1% Triton X-100 served as the positive control. Fluorescence intensity (excitation: 540 nm; emission: 621 nm) was monitored continuously for 70 min in a black polystyrene microtiter plate using a Cytation 5 cell imaging multimode reader (BioTek, Winooski, VT, USA).

### Bacterial membrane potential assay

Changes in membrane potential were evaluated using the voltage-sensitive fluorescent probe DiBAC_4_(3). An exponential-phase culture of *S. aureus* YUSA145 was dispensed into a black polystyrene microtiter plate. DiBAC_4_(3) was added to a final concentration of 1 μM, and the plate was incubated in the dark at 37°C for 20 min. The bacterial suspension was then treated with PFM01 at final concentrations of 12.5, 25, and 50 μM (with a final DMSO concentration of 0.5%, v/v). Treatment with 0.1% Triton X-100 was used as a positive control. Fluorescence intensity (excitation: 492 nm; emission: 515 nm) was recorded continuously for 70 min using a Cytation 5 cell imaging multimode reader (BioTek, Winooski, VT, USA).

### Assessment of exogenous phospholipids on PFM01 activity

The potential interaction between PFM01 and key bacterial membrane phospholipids was investigated by broth microdilution checkerboard assays. Phosphatidylglycerol (PG), phosphatidylcholine (PC), phosphatidylethanolamine (PE), and cardiolipin (CL) were individually tested for their ability to antagonize the antibacterial activity of PFM01 against *S. aureus* strain YUSA145.

Briefly, serial dilutions of PFM01 were prepared in CAMHB within a 96-well round-bottom microplate to final concentrations ranging from 200 to 1.56 μM. Each well subsequently received a defined concentration of one of the four phospholipids at final concentrations up to 128 μg/mL. After pre-incubation of the drug-phospholipid mixtures at 37°C for 30 min, a bacterial suspension of *S. aureus* YUSA145 (approximately 5 × 10^5^ CFU/mL) was added to each well. The plate was then incubated at 37°C for 24 h. The MIC of PFM01 in the presence of each phospholipid was determined visually and compared with the MIC obtained in a control group without phospholipid supplementation. The fold change in MIC was calculated to quantify the modulating effect of each phospholipid.

### Comparative proteomics analysis by LC-MS/MS

Global proteomic profiling was performed using liquid chromatography-tandem mass spectrometry (LC-MS/MS) to investigate the response of *S. aureus* to PFM01 exposure. *S. aureus* strain SA113 was cultured to the logarithmic growth phase and then treated with PFM01 at a concentration of 1× MIC (12.5 μM, with a final DMSO concentration of 0.125%, v/v), or with an equal volume of DMSO as a vehicle control, in TSB at 37°C with shaking (220 rpm) for 4 h. Bacterial cells were harvested by centrifugation (5,000 × *g*, 10 min, 4°C) and washed twice with ice-cold PBS. Total protein was extracted from the cell pellets using RIPA lysis buffer supplemented with a protease inhibitor cocktail (Beyotime, Shanghai, China) and 0.1-mm diameter glass beads, with mechanical disruption performed at 70 Hz. The lysate was clarified by centrifugation (12,000 × *g*, 15 min, 4°C), and the protein concentration in the supernatant was determined using a bicinchoninic acid (BCA) assay kit (Thermo Fisher Scientific, USA). For proteomic sample preparation, proteins were reduced with 10 mM dithiothreitol (DTT) at 70°C for 1h and subsequently alkylated with 50 mM iodoacetamide (IAA) in the dark for 15 minutes. Following desalting and filtration using Amicon Ultra centrifugal filters, proteins were digested with sequencing-grade trypsin (Promega, Madison, WI, USA) at 37°C overnight. The resulting peptides were separated on a C18 reversed-phase analytical column (75 μm × 250 mm, Acclaim PepMap RSLC, 2 μm) and analyzed using a Q Exactive Plus hybrid quadrupole-Orbitrap mass spectrometer (Thermo Fisher Scientific Inc.).

### Induction of PFM01 resistance mutations *in vitro* and Whole-genome sequencing

PFM01-resistant mutants were generated from *S. aureus* strain SA113 under the pressure of PFM01, starting from 1/2 times the MIC, and gradually increasing the induction concentration according to the MIC detected each time, and continuously induced and passaged. For the original strain and the induced strain, the bacterial genome was extracted using a genome extraction kit and sent to Novogene for whole-genome sequencing. The sequencing results were mapped to the genome of the reference sequence on NCBI of the standard strain using Illumina PE150 sequencing data. Use MUMMER comparison software and screen for SNPs (single nucleotide polymorphisms).

### Drug affinity responsive target stability assay (DARTS)

The DARTS assay was performed to identify potential protein targets of PFM01 in *S. aureus* strain SA113. Bacterial cells in the logarithmic growth phase were harvested, washed three times with cold PBS, and resuspended in PBS supplemented with a complete protease inhibitor cocktail. Cell lysis was achieved through mechanical disruption using 0.1-mm glass beads. The lysate was clarified by centrifugation at 12,000 × *g* for 20 min at 4°C, and the protein concentration in the supernatant was determined using a bicinchoninic acid (BCA) assay kit.

Equal aliquots of the protein extract were pre-incubated with either 100 μM PFM01 (with a final DMSO concentration of 1%, v/v) or DMSO as a vehicle control at 4°C for 2 h. Each sample was then subjected to limited proteolysis by adding Pronase at a Pronase-to-protein mass ratio of 1:200, followed by incubation at room temperature for 30 min. The proteolysis reaction was terminated by adding SDS loading buffer and heating at 95°C for 10 min. Protein samples were separated by SDS-PAGE. Protein bands that showed differential stability between the PFM01-treated and control groups were excised from the gel and identified by LC-MS/MS. Proteins exhibiting a ≥ 1.5-fold increase in band intensity (*P* < 0.05) upon PFM01 treatment were considered candidate binding targets.

### Molecular docking analysis

The three-dimensional structure of the target protein TarD (UniProt ID: Q2G2 X 2) was obtained from the UniProt database (https://www.uniprot.org/) in PDB format, and the three-dimensional structure of the PFM01 was obtained from the PubChem database (https://pubchem.ncbi.nlm.nih.gov/) in SDF format. Protein-ligand blind docking was performed using the CB-Dock2 server (https://cadd.labshare.cn/cb-dock2/). The protein PDB file and ligand SDF file were uploaded to the server, which automatically performed binding cavity detection, AutoDock Vina docking, and homologous template optimization. Default parameters were used (detecting the top five largest cavities), and the Vina score was used as the binding affinity evaluation metric (a lower score indicates stronger binding). The docking results were preliminarily analyzed using the server’s built-in 3D viewer, further displaying interactions, such as hydrogen bonds and hydrophobic interactions between the ligand and key residues of the protein (43).

### Statistical analysis

All statistical analyses were conducted using GraphPad Prism Software (version 8.0). Data are presented as the mean ± SD. Unless otherwise specified, *P*-values were calculated using ANOVA, with significance levels denoted as *P < 0.05, **P < 0.01, ***P < 0.001, and ****P < 0.0001.

## Data Availability

The proteomics data generated by mass spectrometry have been submitted to the iProX Consortium, with the assigned data set identifier IPX0010731000.

## SUPPLEMENTAL MATERIAL

Figure S1 spectrum.01372-26-s0001.tif Figure S1

Supplemental tables and figure spectrum.01372-26-s0002.docx Tables S1 to S3 and Figure S1.

ASM does not own the copyrights to Supplemental Material that may be linked to, or accessed through, an article. The authors have granted ASM a non-exclusive, world-wide license to publish the Supplemental Material files. Please contact the corresponding author directly for reuse.
