# Supplementary material for: Repurposing PFM01 as a novel antibacterial agent against Staphylococcus aureus
Source: Microbiol Spectr. 2026 Oct 1;14(11):e01372-26. doi: 10.1128/spectrum.01372-26 (PMC13729225; doi:10.1128/spectrum.01372-26)
Supplement: Supplementary file 1 [file spectrum.01372-26-s0002.docx]

**Table S1 Proteomic analysis of *S. aureus* SA113 treated with PFM01**

| **Class** | **Uniprot ID** |
| --- | --- |
| **Up-regulated** | SAOUHSC_02866, HrtA, HrtB, SAOUHSC_00690, ArgG, SAOUHSC_00914, RplP, SAOUHSC_01991, RplA, Rnr, SAOUHSC_02867, SAOUHSC_01854, MnmE, FabZ, SAOUHSC_01990, ArgH, SAOUHSC_00228, SasG, SAOUHSC_02099, SAOUHSC_01394, CorA, DapB, SAOUHSC_00548, Mfd, SAOUHSC_02769, CshB, Asd, SAOUHSC_02849, IsaB, SAOUHSC_02131, SAOUHSC_02139, SAOUHSC_00732, SAOUHSC_00284, SAOUHSC_02161, AldA, SAOUHSC_02430, Mgt, SAOUHSC_00167, SAOUHSC_01679, AldH1, SAOUHSC_00961, SAOUHSC_00870, SplB, SAOUHSC_02525, SAOUHSC_01613, SAOUHSC_01382, MraZ, SAOUHSC_02760, RplB, EssC, CshA, DapH, UgtP, SAOUHSC_00174, SAOUHSC_00749, SAOUHSC_00074, clfA, SAOUHSC_00902, SAOUHSC_02003 |
| **Down-regulated** | SAOUHSC_02035, SspP, SsaA2, SAOUHSC_00310, SAOUHSC_00088, PflA, RpsP, SAOUHSC_02044, SAOUHSC_01595, SAOUHSC_01297, SAOUHSC_02896, SAOUHSC_00818, SAOUHSC_02064, SAOUHSC_00129, SAOUHSC_02085, SAOUHSC_00170, SAOUHSC_00941, Spx, RpmG2, SAOUHSC_02016, SAOUHSC_02751, SAOUHSC_02093, SAOUHSC_00312, RplQ, SAOUHSC_02022, Sbi, SAOUHSC_00830, RpmA, SAOUHSC_01429, SAOUHSC_02227, SAOUHSC_00873, SAOUHSC_02845, SAOUHSC_02073, TrxA, SAOUHSC_01468, SceD, SAOUHSC_01464, RpsZ, RpoY, SAOUHSC_00918, SAOUHSC_02283, ArcR, NanE, SAOUHSC_00827, SAOUHSC_02013, SAOUHSC_00135, IsaA, SAOUHSC_02791, PflB, SAOUHSC_01443, PyrC, SAOUHSC_00176, SAOUHSC_01114, SAOUHSC_02742, SAOUHSC_00897, SAOUHSC_00946, SAOUHSC_02217, SAOUHSC_00834, SAOUHSC_02010, SAOUHSC_03022, SAOUHSC_02042, SAOUHSC_01405, SAOUHSC_00671, SAOUHSC_02747, SAOUHSC_00215, SAOUHSC_02544, SAOUHSC_01130, SAOUHSC_02129, SAOUHSC_00061, SAOUHSC_00705, PurH, SAOUHSC_02239, SAOUHSC_02901, SAOUHSC_00975, lrgA, SAOUHSC_00874, SAOUHSC_00357, SAOUHSC_00429, RpmB, RpsU, SAOUHSC_01295, GcvH, SAOUHSC_00481, SAOUHSC_00890, SAOUHSC_01525, SAOUHSC_02909, LtaS, SAOUHSC_02043, ArsC, CspA, SAOUHSC_02321, PyrB, PurE, SAOUHSC_02576, InfA, SAOUHSC_00003, IlvC, PyrR, SAOUHSC_02683, SAOUHSC_01044, SAOUHSC_02886, SAOUHSC_01700, SAOUHSC_01463, SAOUHSC_00371, SAOUHSC_01134, SAOUHSC_01730, PurD, SAOUHSC_01028, PurM, PurF, PurQ, RpmF, DltC, SAOUHSC_00330, PurC, RsbV, SAOUHSC_00680, CopZ, SAOUHSC_02781, SAOUHSC_01860, Purl, SAOUHSC_00951, SAOUHSC_01110, SAOUHSC_01245, AcpP, SAOUHSC_02171, SAOUHSC_01438, SAOUHSC_01949, AcyP, PurS, SAOUHSC_01244, SAOUHSC_02538, ArcC2, SAOUHSC_00041, PurK, PurA, SAOUHSC_00755, SAOUHSC_02014, SAOUHSC_00202, SAOUHSC_01504, SAOUHSC_02402, PurN, SAOUHSC_01349, SsaA, SAOUHSC_00311, HysA, SAOUHSC_02282, SAOUHSC_01874, SAOUHSC_02827, ArgF, SAOUHSC_00819, ArcA, SAOUHSC_01542, SAOUHSC_03045 |

**Table S2 MIC of PFM01 against *S. aureus* SA113 wild-type and *mprF*/*clpX* knockout mutants.**

| **Strains** | **MIC (μM)** |
| --- | --- |
| SA113 | 12.5 |
| △*mprF* | 12.5 |
| △*clpX* | 12.5 |

**Table S3 DARTS-based screening of PFM01-interacting proteins in *S.aureus* SA113.**

| **Class** | **Uniprot ID** |
| --- | --- |
| **Up-regulated** | TarD, MurA.1, MurA, FtnA, HemL1, TarK, AzoR, GlmM, RpsQ, Rho, SAOUHSC_00909, SAOUHSC_01594, SAOUHSC_02820, SAOUHSC_01282, MenC, AldA, SAOUHSC_00669, PepT, Der, SAOUHSC_00462, FemX, SAOUHSC_01198, SAOUHSC_00878, ArgF.1, RpsC, Frr, Gmk, GlpD, Prs |
| **Down-regulated** | SAOUHSC_01837, SAOUHSC_01365, SAOUHSC_01193, SAOUHSC_00634, SAOUHSC_01392, SAOUHSC_00808, RplI, SAOUHSC_02158, FabZ, SAOUHSC_01721, SAOUHSC_01675, SAOUHSC_01184, SAOUHSC_02845, SAOUHSC_02648, SAOUHSC_02830 |

**Fig. S1** **Growth curves of *S. aureus* strains treated with PFM01 or daptomycin.**

**(a-c)** Growth curves of the wild-type SA113 **(a)**, *mprF* knockout **(b)**, and *clpX* knockout **(c)** strains treated with PFM01 at 1/8, 1/4, 1/2, and 1×MIC, respectively. **(d-f)** Growth curves of the same strains treated with daptomycin (**d**: wild-type SA113, **e**: *mprF* knockout, **f**: *clpX* knockout). Growth curves of untreated controls for each strain are also included. *n* = 3, data are presented as mean ± SD.
